# Asymmetric Interoperability as a Strategy Among Provider Group Health Information Exchange: Directional Analysis

**DOI:** 10.2196/43127

**Published:** 2023-04-06

**Authors:** Rohin Rathin Shah, Joseph Peter Bailey

**Affiliations:** 1 The Robert H. Smith School of Business University of Maryland College Park, MD United States; 2 Poolesville High School Poolesville, MD United States

**Keywords:** health information exchange, quality payment program, electronic health records, electronic referrals, medical informatics, technology adoption, health information interoperability

## Abstract

**Background:**

High levels of seamless, bidirectional health information exchange continue to be broadly limited among provider groups despite the vast array of benefits that interoperability entails for patient care and the many persistent efforts across the health care ecosystem directed at advancing interoperability. As provider groups seek to act in their strategic best interests, they are often interoperable and exchange information in certain directions but not others, leading to the formation of asymmetries.

**Objective:**

We aimed to examine the correlation at the provider group level between the distinct directions of interoperability with regard to sending health information and receiving health information, to describe how this correlation varies across provider group types and provider group sizes, and to analyze the symmetries and asymmetries that arise in the exchange of patient health information across the health care ecosystem as a result.

**Methods:**

We used data from the Centers for Medicare & Medicaid Services (CMS), which included interoperability performance information for 2033 provider groups within the Quality Payment Program Merit-based Incentive Payment System and maintained distinct performance measures for sending health information and receiving health information. In addition to compiling descriptive statistics, we also conducted a cluster analysis to identify differences among provider groups—particularly with respect to symmetric versus asymmetric interoperability.

**Results:**

We found that the examined directions of interoperability—sending health information and receiving health information—have relatively low bivariate correlation (0.4147) with a significant number of observations exhibiting asymmetric interoperability (42.5%). Primary care providers are generally more likely to exchange information asymmetrically than specialty providers, being more inclined to receive health information than to send health information. Finally, we found that larger provider groups are significantly less likely to be bidirectionally interoperable than smaller groups, although both are asymmetrically interoperable at similar rates.

**Conclusions:**

The adoption of interoperability by provider groups is more nuanced than traditionally considered and should not be seen as a binary determination (ie, to be interoperable or not). Asymmetric interoperability—and its pervasive presence among provider groups—reiterates how the manner in which provider groups exchange patient health information is a strategic choice and may pose similar implications and potential harms as the practice of information blocking has in the past. Differences in the operational paradigms among provider groups of varying types and sizes may explain their varying extents of health information exchange for sending and receiving health information. There continues to remain substantial room for improvement on the path to achieving a fully interoperable health care ecosystem, and future policy efforts directed at advancing interoperability should consider the practice of being asymmetrically interoperable among provider groups.

## Introduction

### Background

Achieving a fully interoperable health care ecosystem—spanning patients, providers, payers, and public health organizations—in the era of increased use of information systems is a fundamental building block of providing the best possible patient care. Health information exchange (HIE), referenced as a form of interoperability, is the electronic transfer of patient health information in a standardized and secure manner [1,2]. In care settings, the benefits of HIE are multifold and include improved patient care outcomes, lowered costs to seek care, reduced imaging, and fewer redundant procedures [3-6], underscoring the importance of HIE. Clinicians, policy makers, researchers, and patients recognize HIE as a way to deliver higher quality and higher value health care [7] and remedy the highly fragmented state of the health care ecosystem as it currently stands [8].

In practice, information exchange is often not fully integrated between providers because of challenges with interoperability, including disparate health information systems, varying patient data collection and storage practices, and organizational barriers, among others. On the basis of the mandates from the Office of the National Coordinator for Health Information Technology (ONC) stemming from the Health Information Technology for Economic and Clinical Health (HITECH) Act of 2009 along with the 21st Century Cures Act of 2016, the vast majority of providers use Certified Electronic Health Record Technology (CEHRT) technology [9], although only 34% of office-based physicians fully engaged in HIE in a bidirectional manner in 2019 [10]. The rest either did not engage in HIE to any extent or did so in a manner that favored certain directions or forms of HIE over others by only exchanging information in that direction, or under that form, of HIE. These limited rates of adoption and use for HIE persist, despite the established aforementioned benefits of HIE for patient care along with the advent and implementation of several provider incentive programs over the past several years that have been directed at increasing HIE adoption and use. These incentive programs include the promoting interoperability performance category of the Quality Payment Program (QPP) Merit-based Incentive Payment System (MIPS) established in 2017 [11] along with the separate Promoting Interoperability Programs (previously the Electronic Health Record [EHR] Incentive Programs) established in 2011 [12], both of which are under the auspices of the Centers for Medicare & Medicaid Services (CMS).

### Objectives

As policies continue to evolve to help promote interoperability, it is critical to understand the degree to which interoperability is asymmetric among different providers. As prominent, ecosystem-wide standards that govern how health information is stored and exchanged, such as Health Level 7 (HL7) Fast Healthcare Interoperability Resources (FHIR), have matured and become more widely adopted and advanced in recent years, achieving ecosystem-wide interoperability and HIE use appears to have become somewhat less of a technological problem and relatively more of a problem defined by providers, perhaps, seeking to act strategically, based on what would be the most advantageous and appropriate for their specific practice [13], leading to different providers adopting and using HIE in different ways. Accordingly, we seek to examine how certain practice characteristics that may guide those strategic decisions regarding HIE—such as being a primary care provider (PCP) versus a specialty provider along with group size—influence to what extent provider groups engage in HIE in a symmetric or asymmetric manner. We defined engaging in HIE in a symmetric manner as not favoring one direction of electronic information exchange over the other; that is, having similar rates of HIE use for sending and receiving health information. We defined engaging in HIE in an asymmetric manner as favoring one direction of electronic information exchange over the other and being more inclined to exchange information in that direction; that is, having higher rates of HIE use for sending health information as opposed to lower rates of HIE use for receiving health information or vice versa. We did not make any distinction for how patient health information was exchanged electronically, as long as it did occur electronically and CEHRT was used based on the data that we used in our study. Previous work has explored what factors affect HIE use specifically for sending health information with electronic referrals [14], the barriers to HIE adoption that providers face [15,16], and the impact of HIE adoption and use [4-6]. However, limited work has investigated HIE use at the group or practice level in a manner that distinctively separates the directions of exchange for providers (sending vs receiving) to examine and compare the presence of symmetries and asymmetries in HIE use among certain types of provider groups as opposed to others, which was the aim of this study.

In this study, we leveraged data available from the CMS, the largest payer for health care services in the United States, to understand symmetric and asymmetric interoperability and HIE use among providers. We are interested in evaluating the correlation (or lack thereof) between HIE use for sending health information and receiving health information. Furthermore, we are also interested in examining how this correlation and relationship between HIE use for sending health information and receiving health information, respectively, varies based on provider group type and size along with the symmetries and asymmetries that may form as a result.

## Methods

### Ethical Considerations

The research presented in this study uses archival data publicly available from the CMS and does not use human participants or any personally identifiable information. Therefore, ethics approval was not required for this study.

### Data

For this study, we used the CMS Provider Data Catalog data [17], which largely mirror Medicare Care Compare [18,19], a directory tool developed by the CMS to facilitate provider quality transparency and empower patients to seek care from providers who deliver high-quality care. The CMS Provider Data Catalog includes a variety of data sets that provide insight about providers, including demographic characteristics and responses to performance measures and attestations as part of certain incentive programs, among other information. Considering that 2 of the key, overarching purposes of HIE in empowering patients and enhancing the quality of care that patients receive directly align with that of the Provider Data Catalog, we combined data from 2 data sets within this catalog for use within our study.

Within the CMS Provider Data Catalog, we focused on data available from the QPP MIPS to measure provider HIE use and analyze symmetries and asymmetries. QPP MIPS is a CMS effort focused on increasing the delivery of high-quality and high-value health care by providing providers with positive, neutral, or negative payment adjustments based on their responses to certain performance measures and attestations each year. Medicare and Medicaid clinicians are required to participate in QPP MIPS if they are eligible; however, they are able to choose whether to do so individually, as part of a group, or both individually and as part of a group simultaneously. We specifically drew upon the *QPP Doctors and Clinicians Performance Year (PY) 2020 Group Public Reporting: MIPS Measures and Attestations* file [20], which includes performance information specific to group participation in QPP MIPS. Although previous work has used QPP MIPS data to ascertain predictors of provider performance for providers of differing specialties [21,22] and determine how to optimally allocate health information technology resources to maximize performance [23], we present a novel use of these data in exploring performance specifically as they relate to HIE and interoperability. Although several other sources of data also provide insight into provider HIE and interoperability, we selected this data set for several reasons. Namely, this data set includes separate performance measure data for both sending health information and receiving health information, which is essential to examining where symmetries and asymmetries may exist among provider HIE (the central focus of this study). This data set also has a relatively higher degree of timeliness with data from 2020, as the latest versions of most other data sets include data from 2019 or before. Finally, this data set also focuses specifically on the performance of provider groups as a coherent whole (as opposed to individual clinicians), which is markedly more relevant when analyzing HIE, as achieving interpractice interoperability poses many more barriers and is far more complex compared with achieving intrapractice interoperability.

In addition, within the Provider Data Catalog, to determine provider group demographic characteristics, we used the *National Downloadable File* (NDF) [24]. This data set includes information about all providers—not just limited to those who participated in QPP MIPS, whether individually or as part of a group—who have complete, current, and approved records in the CMS Provider Enrollment, Chain, and Ownership System, where individual clinicians manage their enrollment as Medicare and Medicaid providers.

The aforementioned QPP MIPS performance data set is organized at the provider group level with Organization Provider Enrollment, Chain, and Ownership System Associate Control (PAC) ID as the unique identifier, whereas the NDF is organized at the individual clinician level with National Provider Identifier as the unique identifier. For individual clinicians part of a provider group, the NDF also includes a field for Organization PAC ID to identify which provider group those individual clinicians are a part of. Consequently, as both data sets are organized at different levels with different scopes, to reconcile between them, all individual clinician records in the NDF without a populated Organization PAC ID that was also found in the QPP MIPS performance data set were discarded, and the remaining records were sorted according to Organization PAC ID to group individual clinicians from the same provider group together.

### Measures

The QPP MIPS performance data set includes measures and attestations that fall into 1 of 4 categories: quality, promoting interoperability, improvement activities, and cost. Each category comprises a certain percentage of provider groups’ final scores, which determines the payment adjustment they receive from their participation in QPP MIPS. To separately measure provider group HIE use for sending and receiving health information, 2 measures were selected from the promoting interoperability category (30% of final scores): *Support Electronic Referral Loops By Sending Health Information* (hereinafter known as the *sending metric*) and *Support Electronic Referral Loops By Receiving and Incorporating Health Information* (hereinafter known as the *receiving metric*). For each of these measures, provider group performance rates can plausibly range from 0 to 100 and are calculated based on the percentage of all referrals and transitions of care in which HIE was leveraged to send (receive) patient health information during the reporting period, a self-selected 90-day period during the calendar year. For the sending metric, the performance rate denominator is the total number of referrals and transitions of care sent or ordered by a given provider group through any and all means (whether electronic or nonelectronic) during the reporting period, and the performance rate numerator is the number of referrals and transitions of care from the denominator for which patient health information was appropriately sent electronically through HIE [25]. For a referral or transition of care to count toward the performance rate numerator for the sending metric, the information exchanged must include a summary of care record prepared using CEHRT, which must detail, if known, the following information about a patient: name, demographic information, smoking status, current problem list, current medication list, current medication allergy list, laboratory tests, laboratory results, vital signs, procedures, care team members, immunizations, unique device identifiers for implantable devices, care plan, referring or transitioning clinician’s name and office contact information, encounter diagnosis, functional status, and reason for referral. For the receiving metric, the performance rate denominator is the total number of referrals and transitions of care received electronically using CEHRT by a given provider group during the performance period for patients never before encountered, and the performance rate numerator is the number of referrals and transitions of care from the denominator for which full reconciliation was completed to natively incorporate all received patient information in the provider group’s EHR system [26]. For a referral or transition to count toward the performance rate numerator for the receiving metric, the following clinical information about a patient must be reconciled using CEHRT: current medication list, current medication allergy list, and current problem list. For each of these measures, exclusions could be claimed by provider groups either voluntarily or by sending (receiving) <100 patient referrals during the reporting period (ie, having a denominator value <100). In the event that an exclusion is claimed for a specific measure by a given provider group, the points associated with that measure are redistributed to another measure in the promoting interoperability category. Among the approximately 20,149 provider groups represented in the QPP MIPS data set, 4583 provider groups reported data for the sending metric and 3430 provider groups reported data for the receiving metric, with 3619 provider groups reporting data for just one measure and 2197 provider groups reporting data for both measures. Only provider groups that reported data for both measures were included in the sample and the rest were excluded.

The NDF was used to determine whether each provider group should be classified as a PCP or as a specialist. To do so, for each provider group in the sample, we took the mode of the *Primary Specialty* field for all the individual clinician records in the NDF from the same provider group. Provider groups with family practice, internal medicine, hospitalist, general practice, or pediatric medicine as their mode were classified as PCPs. All other provider groups were classified as specialists. Within the NDF, the records of individual clinicians with specialties that were deemed to be agnostic to practices of differing specialties were removed to not skew the classification. For example, both pediatric practices and gynecology practices may have nurse practitioners that are more abundant than physicians. Therefore, excluding the records of nurse practitioners makes it easier to accurately determine the specialty of those practices. These excluded specialties included nurse practitioner, physician assistant, anesthesiology assistant, certified clinical nurse specialist, certified registered nurse anesthetist, certified nurse midwife, and clinical social worker.

The NDF was additionally used to determine the size of each provider group. To do so, for each provider group in the sample, we verified that all the records for individual clinicians in the NDF from the same provider group had the same value for the *Number of Group Members* field and subsequently took that value. Then, we classified provider groups by quartile based on group size with the first quartile including the smallest provider groups and the fourth quartile including the largest provider groups.

The final sample included all provider groups who reported data for both the sending metric and the receiving metric and were also included in the NDF through the listed group affiliations of individual clinicians. A total of 2197 provider groups reported data for both the sending metric and the receiving metric. From those provider groups, 2033 had referenceable records in the NDF, forming our sample. We eliminated the 164 provider groups that did not have referenceable records in the NDF, representing <8% of the potential sample. We examined these excluded observations and found no statistical difference between the characteristics of the observations that were excluded and the 2033 observations that constituted the sample that we used for our analysis.

### Analysis

From our sample, we calculated the bivariate correlation between the sending metric and the receiving metric to examine the relationship that exists between both variables. We also created kernel density estimate plots to analyze and compare the distributions of the sending metric and the receiving metric for provider groups when not segmented, when segmented by PCP or specialist classification, and when segmented by group size quartile.

Because of the nonnormal distribution of the data, statistical procedures that presume normality as an assumption were not used. Instead, to pinpoint trends within the data, we conducted k-means clustering both when the sample was not segmented and when the sample was segmented based on PCP or specialist classification and provider group size. The optimal number of clusters for each clustering plot was determined using the elbow method by identifying the number of clusters after which the reduction in the model’s inertia from adding an additional cluster largely became insignificant. To investigate the presence of symmetries and asymmetries, we compared the sparsity or density of certain clusters as opposed to other clusters both within the same clustering plot and other clustering plots from the same segmentation.

## Results

### Overview

From the clustering approach we used, we defined 4 clusters of varying extents of HIE use among provider groups for sending health information and receiving health information. As shown in the columns in Table 1, we generated 4 clusters (high send and high receive, high send and low receive, low send and high receive, and low send and low receive) for each segmentation of the sample. As shown in the rows in Table 1, our first analysis was done without segmentation using our full sample of 2033 provider groups. Our subsequent 2 analyses were done after segmenting our sample of provider groups based on PCP or specialist classification and based on group size by quartile.

**Table 1:** Number of observations found within each of the 4 clusters for health information exchange use among provider groups.

		Clusters					Correlation between send and receive		Total^a^, n
		1: High send and high receive, n (%)	2: High send and low receive, n (%)	3: Low send and high receive, n (%)	4: Low send and low receive, n (%)				
Overall		433 (21.3)	311 (15.3)	553 (27.2)	736 (36.2)	0.4147		2033	
**Provider group type—PCP^b^ or specialist classification**									
	PCP	104 (10.9)	158 (16.6)	298 (31.3)	391 (41.1)	0.2962		951	
	Specialist	329 (30.4)	153 (14.1)	255 (23.6)	345 (31.9)	0.4620		1082	
**Provider group size—quartile [range of the number of members)^c^**									
	Q1 [2-8)	146 (29.5)	77 (15.6)	142 (28.7)	130 (26.3)	0.3715		495	
	Q2 [8-35)	162 (31.2)	62 (11.9)	140 (26.9)	156 (30)	0.4945		520	
	Q3 [35-132)	93 (18.3)	83 (16.3)	141 (27.8)	191 (37.6)	0.3560		508	
	Q4 [132-4958]	32 (6.3)	89 (17.5)	130 (25.5)	259 (50.8)	0.2598		510	

^a^The sum of percentages in a given row may not equal 100% because of intermediate rounding.

^b^PCP: primary care provider.

^c^The range of the number of group members that defines each quartile has an inclusive lower bound and an exclusive upper bound, except that of the fourth quartile, which has both an inclusive lower bound and an inclusive upper bound.

### Distribution of Sending Metric and Receiving Metric

**Figure 1:**
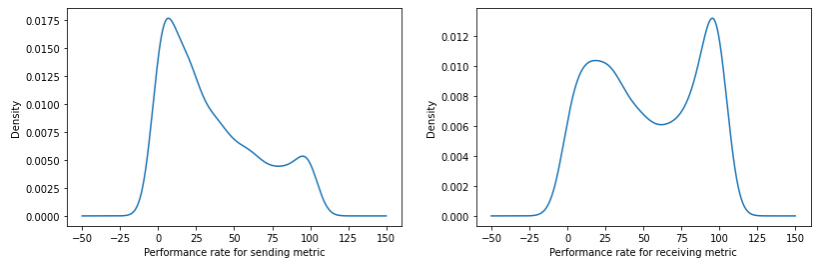
Kernel density estimation plots without segmentation for the sending metric (left) and the receiving metric (right).

When comparing the performance distribution of the sending metric and the receiving metric when our sample was not segmented (Figure 1), we found that provider groups generally exhibit higher performance rates for receiving health information and appear to be more inclined to receive and reconcile health information (ie, conceivably acquiring new patients and growing their practice), compared with sending health information (ie, conceivably losing patients, particularly among provider groups of the same specialty or PCP type who serve similar patients). For the sending metric, the vast majority of provider groups use HIE to send health information less than half of the time. On the other hand, for the receiving metric, although a sizable proportion of provider groups do exhibit marginal rates of HIE use, an even larger proportion of provider groups exhibit substantially higher rates of HIE use to receive and reconcile health information.

**Figure 2:**
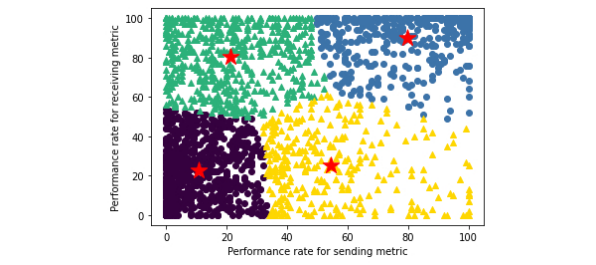
K-means clustering plot with centroids for sending metric versus receiving metric performance rates among provider groups without segmentation.

From the 4 distinct clusters we generated to represent varying levels of HIE use when our sample was not segmented (Figure 2), we found that 42.5% (864/2033) of provider groups in our sample used HIE in an asymmetric manner (Textbox 1), which most commonly occurred by having high performance for receiving health information but low performance for sending health information. The most provider groups with 36.2% (736/2033) of our sample were concentrated in the cluster representative of low levels of performance for both sending and receiving health information, whereas the fewest provider groups with 15.3% (311/2033) of our sample were concentrated in the cluster representative of high levels of performance for sending health information but low levels of performance for receiving health information. Finally, we found a low level of bivariate correlation of 0.4147 between the sending metric and the receiving metric.

**Key for k-means clustering (k=4) in Figure 2.**

Circular markers and triangular markers are indicative of symmetric and asymmetric health information exchange (HIE) use, respectively.

- 1: blue (high send and high receive)—indicative of symmetric HIE use in having high performance for both sending and receiving health information (n=433).
- 2: yellow (high send and low receive)—indicative of asymmetric HIE use in having high performance for sending but low performance for receiving health information (n=311).
- 3: green (low send and high receive)—indicative of asymmetric HIE use in having low performance for sending but high performance for receiving health information (n=553).
- 4: purple (low send and low receive)—indicative of symmetric HIE use in having low performance for both sending and receiving health information (n=736).

### PCPs Versus Specialists

**Figure 3:**
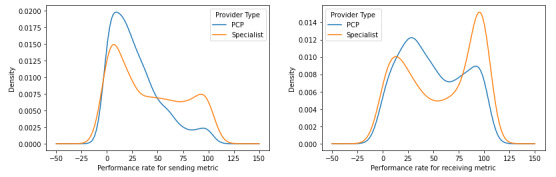
Kernel density estimation metric plots segmented by provider group primary care provider (PCP) or specialist classification for the sending metric (left) and the receiving metric (right).

We found that specialists tend to exhibit markedly higher rates of HIE use than PCPs for both sending and receiving health information (Figure 3). However, at the same time, we also found that, although the extent of use and performance varies among both PCPs and specialists, the general distribution of performance is largely the same with the sending metric being right skewed and the receiving metric being bimodal. Finally, consistent with a similar trend when provider groups are not segmented, both PCPs and specialists exhibit higher rates of HIE use for receiving health information as opposed to sending health information.

**Figure 4:**
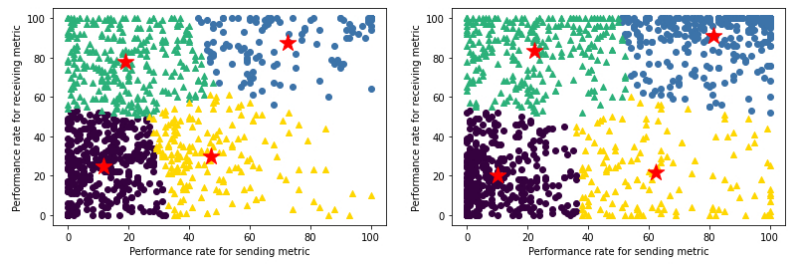
K-means clustering plots with centroids for sending metric versus receiving metric performance rates among primary care provider groups (left) and specialty provider groups (right).

PCP groups tend to exhibit relatively higher rates of asymmetric HIE use compared with specialty provider groups, as 47.9% (456/951) of PCP groups used HIE in an asymmetric manner, whereas 37.7% (408/1082) of specialty provider groups used HIE in an asymmetric manner (Textbox 2). In addition, specialty provider groups tend to have substantially higher rates of HIE use, as the upper-right cluster (Figure 4), representative of high performance for both sending and receiving health information, is composed of 30.4% (329/1082) of specialty provider groups but only 10.9% (104/951) of PCP groups—a nearly 3-fold disparity.

**Key for k-means clustering (k=4) in Figure 4.**

Circular markers and triangular markers are indicative of symmetric and asymmetric health information exchange (HIE) use, respectively.

- 1: blue (high send and high receive)—indicative of symmetric HIE use in having high performance for both sending and receiving health information (primary care provider [PCP]: n=104 and specialist: n=329).
- 2: yellow (high send and low receive)—indicative of asymmetric HIE use in having high performance for sending but low performance for receiving health information (PCP: n=158 and specialist: n=153).
- 3: green (low send and high receive)—indicative of asymmetric HIE use in having low performance for sending but high performance for receiving health information (PCP: n=298 and specialist: n=255).
- 4: purple (low send and low receive)—indicative of symmetric HIE use in having low performance for both sending and receiving health information (PCP: n=391 and specialist: n=345).

### Provider Group Size

**Figure 5:**
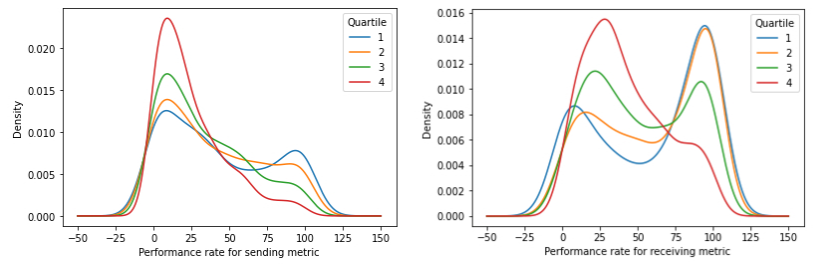
Kernel density estimation plots segmented by quartile based on provider group size for the sending metric (left) and the receiving metric (right).

Larger provider groups, on average, exhibit lower rates of HIE use for both sending and receiving health information, as opposed to smaller provider groups (Figure 5). However, the sending metric and the receiving metric take on different distributions, both inherently and as it relates to growing provider group size with each progressive quartile. With the sending metric, as provider group size increases, the distribution is consistently right skewed and becomes more so as provider group size increases, indicating that most provider groups used HIE relatively marginally to send health information. With the receiving metric, while its distribution does eventually become right skewed as well with the largest provider groups in Q4, its distribution consistently becomes increasingly bimodal from Q1 to Q3, indicating that most provider groups either use HIE for receiving health information minimally or extensively with few provider groups that use HIE moderately. Finally, as is consistent when the sample was not segmented and was segmented by PCP or specialist classification, provider groups of all sizes tend to exhibit higher rates of HIE use for receiving health information compared with sending health information.

**Figure 6:**
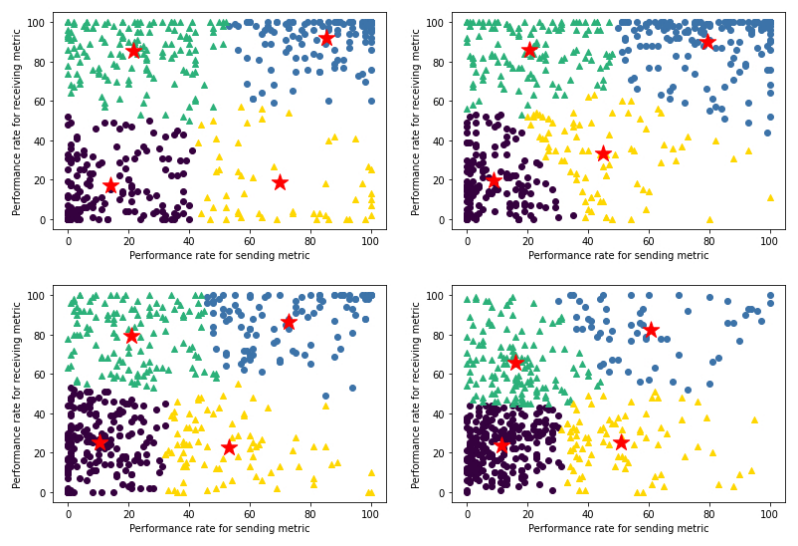
K-means clustering plots with centroids for sending metric versus receiving metric performance rates among provider groups in quartile 1 (upper-left), quartile 2 (upper-right), quartile 3, (bottom-left), and quartile 4 (bottom-right) based on size.

As provider group size increases with each subsequent quartile, the upper-right cluster (Figure 6), representative of high rates of HIE use for both sending and receiving health information, becomes progressively sparse from 29.5% (146/495) of Q1 provider groups concentrated in that cluster to 6.3% (32/510) of Q4 provider groups concentrated in that cluster (Textbox 3)—a nearly 5-fold decrease. Simultaneously, the lower-left cluster, representative of low rates of HIE use for both sending and receiving health information, becomes progressively dense from 26.3% (130/495) of Q1 provider groups concentrated in that cluster to 50.8% (259/510) of Q4 provider groups concentrated in that cluster—a nearly 2-fold increase. The increasing sparsity of the upper-right cluster is commensurate with the increasing density of the lower-left cluster, as the upper-right cluster experiences a decrease of 23.22 percentage points nearly in line with the 24.522 percentage point increase that the lower-left cluster experiences. This is also because the proportion of provider groups who use HIE in an asymmetric manner is relatively constant across all 4 quartiles with 44.2% (219/495) in Q1, 38.8% (202/520) in Q2, 44.1% (224/508) in Q3, and 42.9% (219/510) in Q4.

**Key for k-means clustering (k=4) in Figure 6.**

Circular markers and triangular markers are indicative of symmetric and asymmetric health information exchange (HIE) use, respectively.

- 1: blue (high send and high receive)—indicative of symmetric HIE use in having high performance for both sending and receiving health information (Q1: n=146; Q2: n=162; Q3: n=93; and Q4: n=32).
- 2: yellow (high send and low receive)—indicative of asymmetric HIE use in having high performance for sending but low performance for receiving health information (Q1: n=77; Q2: n=62; Q3: n=83; and Q4: n=89).
- 3: green (low send and high receive)—indicative of asymmetric HIE use in having low performance for sending but high performance for receiving health information (Q1: n=142; Q2: n=140; Q3: n=141; and Q4: n=130).
- 4: purple (low send and low receive)—indicative of symmetric HIE use in having low performance for both sending and receiving health information (Q1: n=130; Q2: n=156; Q3: n=191; and Q4: n=259).

## Discussion

### Overview

We examined interoperability and HIE measures for sending and receiving health information from the CMS QPP MIPS data set to better understand the manner in which provider groups exchange health information. We discovered several important insights with respect to how interoperable providers are in practice with 3 principal findings, surrounding the limited correlation between the directions of interoperability (0.4147). We found the resulting asymmetries in the exchange of patient health information from this limited correlation to be widespread across the health care ecosystem with upward of 2 in 5 provider groups being asymmetrically interoperable. This trend had significant variation among provider groups of differing types (PCPs vs specialty providers), but this trend generally had limited variation among provider groups of differing sizes.

### Principal Findings

Our first finding is that a sizable proportion of provider groups (42.5%) had asymmetric levels of interoperability in favoring one direction of exchange over another. Although information system adoption by providers may be considered a discrete choice (ie, which EHR system to adopt), the actual implementation of such systems is far more nuanced in practice when considering interoperability, as the adoption of HIE capabilities by using a given system (ie, being able to exchange information symmetrically) is distinct from the use of interoperability capabilities by choosing to do so (ie, actually exchanging information—whether symmetrically or asymmetrically). Although we expected provider groups to generally have approximately equal rates of HIE use for sending and receiving information (ie, symmetric use of HIE), we found that this was not the case. This may be a result of the strategic benefits and advantages that may come from overwhelmingly focusing on sending health information or overwhelmingly focusing on receiving health information for provider groups. Although similar findings have been shown in other contexts [27], this is the first study to demonstrate this in the context of HIE. From a geographic standpoint, asymmetric HIE use could also, plausibly, stem from, in part, varying patient consent and privacy regulations across jurisdictions, as it has been shown that a combination of provider incentive programs and patient consent requirements is necessary to increase HIE presence and use [28]. Consequently, the absence of such a combination of policies governing HIE in certain jurisdictions may be a contributing factor for ineffective HIE use—namely, asymmetric interoperability and information exchange, for example, among provider groups in those jurisdictions.

Our second finding is 2-fold; although PCP groups had relatively higher levels of HIE use for receiving health information compared with those of HIE use for sending health information (revealing a prominent asymmetry), specialty provider groups had markedly higher rates of HIE use for both sending and receiving health information overall compared with those of PCP groups. In comparison with that of specialty provider groups, PCP groups may have lower rates of HIE use because of “differences in the centrality of PCPs and specialists in provider referral networks” [14]. Specialty provider groups plausibly seek to maintain as many connections as possible to PCP groups via HIE because provider HIE adoption has been proven to increase the number of referrals received [29], and specialists often prominently rely on referrals from PCPs for new patient acquisition. In turn, this compels specialty provider groups to maintain high rates of HIE use for both sending health information and receiving health information, as it allows them to not only acquire new patients in a more effective and efficient manner but also better participate in these referral networks, should they need to refer a patient to a provider of another specialty or subspecialty, or close the referral loop by returning a given patient’s medical record back to the referring PCP. Accordingly, specialty provider groups become very good at both sending and receiving health information—even in connection to PCP groups with idiosyncratic information systems. This is in line with previous findings that have shown that, surrounding networks effects for HIE use and adoption, PCPs exert greater influence on specialty providers than that of specialty providers on PCPs [30]. By the same token, because of the established centrality of PCPs in provider referral networks, PCP groups have a comparatively limited incentive to maintain high rates of HIE use for both sending health information and receiving health information compared with specialty provider groups. This also may be the result of lower profit from PCPs relative to specialty providers, which may make them less able to invest in additional resources for bidirectional, symmetric HIE. Instead, PCP groups focus their HIE use overwhelmingly on receiving health information, as it is, conceivably, more beneficial to their practice by providing a channel to acquire new patients. In other words, PCP groups and specialty provider groups are incentivized in different ways because of their different, yet intertwined, operational dynamics, leading to different extents of HIE use for sending health information and receiving health information among both. With respect to providers decisions on how to best adopt and use HIE within their practices, it has been shown that providers are often the most influenced by the actions of adjacent providers with whom they share a common population of patients and interact with [31], which is consistent with our findings surrounding the relationship between PCP groups and specialty provider groups participating in a common referral network.

Our third finding is that larger provider groups had markedly lower rates of HIE use for both sending and receiving health information, when compared with smaller provider groups. This finding runs contrary to the results of some previous studies [14], although the difference being that this finding comes because of analyzing HIE use among provider groups for both sending health information and receiving health information, as opposed to just sending health information as examined in previous studies. There are several plausible explanations that could account for this. For example, smaller provider groups may find it more advantageous to innovate by being adopters and more active users of health information technology, including HIE from a technical and operational standpoint, because of their relatively nimble standing and the incentive to grow their practice. In other words, higher rates of HIE use may make it easier for smaller provider groups to acquire new patients by allowing them to receive referrals and begin to build a provider network by sending referrals to, and forming connections with, other providers. On the other hand, larger provider groups often have and are better able to maintain preexisting partnership across expansive health organizations, systems, and networks, which, in turn, may reduce their incentive to be interoperable and engage in HIE, potentially stemming from the benefits those partnerships may yield to begin with in, perhaps, bringing in a steady influx of new patients. In addition, larger provider groups may also find it comparatively unnecessary or more difficult to make use of HIE in a meaningful and comprehensive manner because of their relatively larger footprints. Finally, although we did find that provider group size has notable bearing on the extent to which provider groups are symmetrically interoperable, we also found that provider group size does not appear to be a crucial factor behind the extent to which provider groups are asymmetrically interoperable, as similar proportions of provider groups are asymmetric in their HIE use among both smaller provider groups and larger provider groups.

### Limitations

Although our study benefits from looking at data at the group level, we did not specifically address measures of interoperability and HIE within a provider group. In practice, when clinicians are part of the same group, it is generally easier to exchange health information through a variety of channels, including nonelectronic media and ad hoc processes, considering the relatively close proximity of clinicians and use of similar, if not the same, EHR systems. This may give some additional insight into our finding that larger provider groups have lower levels of interoperability. In other words, their use of intragroup communication and information sharing may be in place of more traditional interoperability and HIE adoption and use. Future research should examine how intragroup interoperability may hinder interoperability between and among provider groups.

Another potential limitation of this research is a result of using the CMS data specifically for the sending metric and the receiving metric. Although prior research has used the CMS data exclusively, the sending metric and the receiving metric are only available for the 2033 provider groups that comprise our sample. This is approximately 10% of all provider groups both included in the QPP MIPS data set and represented in Medicare Care Compare and representative of <5% of providers in the United States. With respect to QPP MIPS, we expect there to be, on average, minimal systemic differences between QPP MIPS provider and non–QPP MIPS providers, apart from their payer mix with the former accepting Medicare and Medicaid. We largely attribute this relatively limited sample size to the fact that provider groups can claim exclusions from reporting performance data for the 2 selected HIE measures fairly easily. Accordingly, it is important not to generalize these findings to the larger context without the important caveat that HIE use data for sending and receiving health care information is more likely to be captured for more advanced provider groups. That said, as the CMS continues to collect data, we hope that our study will provide further support for capturing interoperability and HIE use data for sending and receiving health information.

### Implications

Expanding interoperability to improve the exchange of health information is an increasingly important goal for public health agencies. Through the increased and more efficient exchange of information, clinicians are able to provide higher quality and higher value care to their patients, resulting in better patient care outcomes and lower patient care costs. To this end, achieving a health care ecosystem that is fully interoperable in a symmetric manner remains an important goal, even if it may not always be aligned with the strategic and market realities of provider groups.

Provider groups often have strategic objectives that may prevent them from systematically using HIE for sending and receiving health information. First, some providers may seek to make it easier for new patients to join their practices, while making it difficult for existing patients to leave. Providers may also seek to prevent network leakage, which is when a “patient leaves a health system’s network of care,” as it is estimated to cost health systems millions of dollars each year in lost revenue [32]. This is a finding also from other contexts whereby a firm may invest in a converter to a standard but prevent conversion from a standard [27]. In our research, differences between PCP groups and specialty provider groups are consistent with this asymmetry. Accordingly, effectively measuring provider interoperability and HIE use should not be seen as a binary determination (ie, to be interoperable or to not be interoperable), as this fails to account for the nuance broadly present among provider interoperability and HIE use in actuality—namely in being interoperable and exchanging information in one direction but not the other. In addition, providers have more incentive to build connections with other providers and be interoperable when they have a smaller practice. As a particular specialty or resource that a patient may need is less likely to be available in smaller practices, these practices may seek to maintain high levels of HIE use for both sending and receiving health information.

From a policy perspective, the findings from this study have several implications. Addressing asymmetric HIE use is increasingly important, as pervasive asymmetries in the exchange of health information maintain the potential to have similar detrimental effects on patient care as the practice of information blocking has had in the past, albeit to a somewhat smaller extent, by limiting access to the information that may better inform and guide the care that clinicians provide their patients. With respect to the practice of information blocking, this is a call to action that the government has heeded in the past, as substantial progress has been made in recent years to curb information blocking through the 2016 21st Century Cures Act along with the 2020 ONC Cures Act Final Rule [33,34]. However, the ONC may now want to consider adopting new policies that not only promote high levels of bidirectional, symmetric interoperability but also limit asymmetric interoperability to prevent the same effects of information blocking that negatively affected patient care in the past from recurring. This stems from the fact that current policies may not be adequate—especially when asymmetries are more likely to exist among certain segments of provider groups than others, as shown in this study. Additional incentives specifically targeted at segments of providers who engage in HIE either asymmetrically in any manner or symmetrically with low rates of use for both sending and receiving health information could aid in addressing these concerns. Current efforts being led and carried out by the ONC, such as mandates for open application programming interfaces from the Cures Act Final Rule along with the Trusted Exchange Framework and Common Agreement (TEFCA) and Qualified Health Information Network (QHIN) Technical Framework, could also contribute to improving levels of interoperability among providers for both sending and receiving health information. In addition, to increase their effectiveness, regulatory governance initiatives and programs focused on advancing interoperability and HIE should both take into account the unique, differing strategic objectives and priorities that guide certain types of providers and also effectively measure levels of interoperability and HIE use in a manner that accounts for both symmetric and asymmetric use among providers with the various directions of interoperability. This could, for instance, entail including separate performance measures for sending health information and receiving health information, just as QPP MIPS did in PY 2020. However, in PY 2022, provider groups had the option of simply attesting to engaging in HIE in a bidirectional manner without having to report any quantifiable performance measurements for sending or receiving health information. This makes it increasingly difficult and largely infeasible to examine to what extent providers are interoperable in actuality, determine whether providers are using HIE in a symmetric or asymmetric manner, and pinpoint exactly where asymmetries may exist among providers. In turn, this has the potential to inhibit and limit the effectiveness of efforts to expand interoperability and HIE among providers across the health care ecosystem, underscoring the importance of effective, comprehensive reporting and measurement that is reflective of the nuanced nature of interoperability and HIE use among providers.

The role of government may also go beyond policy making to include additional roles the government has exhibited in other settings, such as the roles of serving as a funder and a purchaser, in addition to the role of serving as a policy maker. As demonstrated by the development of the internet [35], the government can and has promoted interoperability by participating in markets. For example, the government may not only mandate application programming interfaces that result from the Cures Act Final Rule, but they may also provide the necessary resources to invent, build, and maintain these platforms. Through this funding, the government can use its influence to ensure higher levels of bidirectional, symmetric interoperability. The government may also use its influence as a purchaser by only reimbursing claims for providers who maintain bidirectional, symmetric interoperability and are compliant with interoperability standards. By exerting its influence in the market as a funder and purchaser, the government has the ability to ensure accurate reporting and effective, accountable use of interoperability and HIE.

Finally, the government may want to encourage innovation in promoting HIE. Current efforts are focused on patient care and efficiency, which are the early stages of interoperability benefits. With greater amounts of interoperability come more opportunities to benefit from the network effects of increased data exchange. For example, increased data tracking can help determine where a contagious disease might appear across providers, instead of being stuck in a given provider’s EHR system, where it poses no benefit to the broader public health of a population. Furthermore, increased data exchange may help clinicians find patients for a clinical trial or direct patients to a specialist that may provide the best possible care, now that patient data can move more easily among systems. Finally—and with the important caveat that patient data ought to be protected—a larger and more complete corpus of patient data can also be better mined for patient diagnosis, rare disease identification, and health innovation as a whole. For example, high levels of bidirectional, symmetric interoperability can contribute to the development of an infinity, or continuous feedback, loop that can catalyze genome-based innovation by bridging the gap between genomic research and clinical care through privacy-centered access to patient longitudinal health records [36].

Policy makers, providers, researchers, and patients are all focused on interoperability and HIE as a means of better coordinating and improving patient care. Our study examined interoperability and HIE use from the standpoints of both sending and receiving health information. Our findings from this study directly inform efforts to increase interoperability and HIE use by examining directional symmetries and asymmetries in the exchange of information that may come because of certain factors and characteristics and their strategic implications for provider groups. Additional research is needed to examine how asymmetric HIE use can affect the quality of care that patients receive. However, it is clear from our research that the vast majority of provider groups are not achieving high rates of bidirectional, symmetric interoperability and HIE use, and there is quite a bit of room for improvement. Only after this improvement will providers be able to optimally exchange health information with the least amount of friction, delay, and cost to fully realize the benefits of interoperability and HIE for patient care and health innovation to the maximal extent.

## Abbreviations

CEHRT: Certified Electronic Health Record Technology
CMS: Centers for Medicare & Medicaid Services
EHR: electronic health record
FHIR: Fast Healthcare Interoperability Resources
HIE: health information exchange
HITECH: Health Information Technology for Economic and Clinical Health
HL7: Health Level 7
MIPS: Merit-based Incentive Payment System
NDF: National Downloadable File
ONC: Office of the National Coordinator for Health Information Technology
PAC: Provider Enrollment, Chain, and Ownership System Associate Control
PCP: primary care provider
PY: performance year
QHIN: Qualified Health Information Network
QPP: Quality Payment Program
TEFCA: Trusted Exchange Framework and Common Agreement

## Data Availability

All data generated or analyzed during this study are included in the published paper.
